# Correlations between genetically predicted lipid-lowering drug targets and inflammatory bowel disease

**DOI:** 10.1186/s12944-024-02026-y

**Published:** 2024-01-29

**Authors:** Kuiyuan Huang, Shenan Huang, Ming Xiong

**Affiliations:** Department of Gastroenterology, The Second Affiliated Hospital of Nanchang University, Jiangxi, 330000 China

**Keywords:** Mendelian randomization, Inflammatory bowel disease, Lipids

## Abstract

**Background:**

Millions of individuals globally suffer from Inflammatory bowel diseases (IBDs). There is a dearth of large population-based investigations on lipid metabolism and IBDs, and it is unclear whether lipid-lowering drugs target IBDs causally. Consequently, the aim of this study was to investigate the effects of lipid-lowering medication targets on the occurrence and progression of IBDs.

**Methods:**

Among the more than 400,000 participants in the UK Biobank cohort and the more than 170,000 participants in the Global Lipids Genetics Consortium, a total of nine genes linked to lipid-lowering drug targets were obtained (ABCG5/ABCG8, APOB, APOC3, LDLR, LPL, HMGCR, NPC1L1, PCSK9, and PPARA). IBD data were acquired from de Lange et al. (patients/sample size of IBDs: 25042/59957; ulcerative colitis (UC): 12366/45,975; Crohn's disease (CD): 12194/40,266) and the FinnGen cohort (patients/total sample size of IBDs: 4420/176,899; CD: 1520/171,906; UC: 3325/173,711). All four datasets were cross-combined for validation via Mendelian randomization analysis, and potential mediating factors were explored via mediation analysis.

**Results:**

Genetically proxied APOC3 inhibition was related to increased IBD risk (odds ratio (95% confidence interval): 0.87 (0.80–0.95); *P* < 0.01) and UC risk (0.83 (0.73–0.94); *P* < 0.01). IBD and CD risk were reduced by genetic mimicry of LDLR and LPL enhancements, respectively (odds ratioLDLR: 1.18 (1.03–1.36); *P* = 0.018; odds ratioCD: 1.26 (1.11–1.43); *P* = 2.60E-04). Genetically proxied HMGCR inhibition was associated with increased CD risk (0.68 (0.50–0.94); *P* = 0.018). These findings were confirmed through Mendelian analysis of the cross-combination of four separate datasets. APOC3-mediated triglyceride levels may contribute to IBDs partly through mediated triglycerides, *Clostridium *sensu stricto* 1*, *Clostridiaceae* 1, or the *Lachnospiraceae* FCS020 group. LDLR enhancement may contribute to IBDs partly through increasing *Lactobacillaceae*.

**Conclusion:**

Vigilance is required to prevent adverse effects on IBDs (UC) for patients receiving volanesorsen (an antisense oligonucleotide targeting ApoC3 mRNA) and adverse effects on CD for statin users. LPL and LDLR show promise as candidate drug targets for CD and IBD, respectively, with mechanisms that are potentially independent of their lipid-lowering effects.

**Supplementary Information:**

The online version contains supplementary material available at 10.1186/s12944-024-02026-y.

## Background

A substantial burden is placed on health care systems from inflammatory bowel diseases (IBDs), including ulcerative colitis (UC) and Crohn's disease (CD) [1, 2]. IBDs involve a complex mucosal immune response that occurs in the gastrointestinal tract and are characterized by intricate interactions between genetics and environment, leading to a diverse range of clinical, genetic, and molecular manifestations [3]. Currently, the main treatment for IBDs involves the utilization of immunosuppressive agents and immunomodulators. Moreover, new treatment strategies are constantly emerging [4]. Despite the expanding repertoire of therapeutic options available to IBD practitioners, a notable proportion of patients exhibit resistance to these interventions [5].

Dyslipidemia frequently coexists with IBDs [6]. Statins have shown many benefits in animal models of IBDs [7]. Statin use has been shown to reduce new-onset IBD risk in several studies [8, 9]; in addition, targets other than LPL [10], PCSK9 [11, 12] and APOC3 [13] for lipid-lowering drugs, have been found to be altered in IBD patients. However, many studies have not reached consistent conclusions [14]. For example, Peppas et al. [14] suggested that the existing epidemiological data are inadequate to justify employing statins in the prevention or management of IBDs, calling for further studies of superior quality.

There is considerable appeal in studying the effects of modulating lipid pathways in IBDs. First, elucidating the causal pathways implicated in lipid metabolism could enhance the understanding of IBD pathogenesis. Second, elucidating the directional link between cholesterol-reducing medications and IBDs facilitates the implementation of measures to mitigate the risk of IBDs. If the employment of lipid-lowering medications is associated with the incidence of IBDs, then the use of these drugs could aid in the identification of individuals who do not exhibit typical symptoms but are at heightened risk. If lipid-lowering agents reduce the incidence of IBDs, they could be employed as preventive measures for individuals at a heightened risk of dyslipidemia, such as those with a significant familial predisposition. Third, the selection of lipid-lowering agents that have dual effects on lipid levels and IBDs facilitates personalized treatment approaches, particularly in patients with dyslipidemia and severe or refractory IBDs necessitating adjunctive therapy. Finally, drug repurposing entails the process of ascertaining novel therapeutic effects of preexisting [15]. Given the generally established safety of approved drugs, drug repurposing represents a viable and economical method for identifying new treatment strategies for IBDs. By focusing on lipid pathways, the potential for adverse events related to direct immunosuppression can be minimized. Nevertheless, the process of generating supportive evidence poses challenges. The hypothesis of the present study was that lipid-lowering drug targets are causally associated with IBDs and could influence the pathogenesis of IBDs by either promoting or inhibiting their onset.

Traditional pharmacoepidemiologic designs are prone to confounding factors, such as unmeasured characteristics that impact the prescription for lipid-lowering drugs and IBD risk, along with reverse causality (IBDs itself leads to dyslipidemia). The examination of extensive genomic datasets offers a viable avenue for exploring the mechanisms of drug action and the potential for drug repurposing.

Repurposing drugs for various diseases has been guided by genome-wide association studies (GWASs), a notable example of which involved the repurposing of ustekinumab, which was originally used for psoriasis treatment, for CD [15]. Mendelian randomization (MR) mirrors randomized controlled trials in that it uses a genetic surrogate for the variable under scrutiny to examine its causative impact on the result [16]. MR can effectively address certain limitations associated with observational studies, such as uncontrolled confounding factors, the inability to establish causality, and issues related to sample [16].

In addition, the utilization of MR to identify drug targets can effectively illustrate how changes in biomarkers impact long-term health outcomes via targeted therapeutic interventions, predict the efficacy of drugs, and uncover any adverse reactions mediated by specific targets [17].

The novel aspect of this study lies in the systematic exploration of the genetic intersections between lipid metabolism and IBDs. This investigation not only provides a potential perspective on the pathogenesis of IBDs but also opens the door to repurposing lipid-lowering medications as potential treatments for these conditions. This aligns with the objective of personalized medicine, where understanding genetic predispositions can guide more effective and tailored therapeutic strategies.

Therefore, this study applied MR methods to explore the possible effects of targets for cholesterol-reducing drugs on IBDs, drawing on information from various databases.

## Methods

**Fig. 1:**
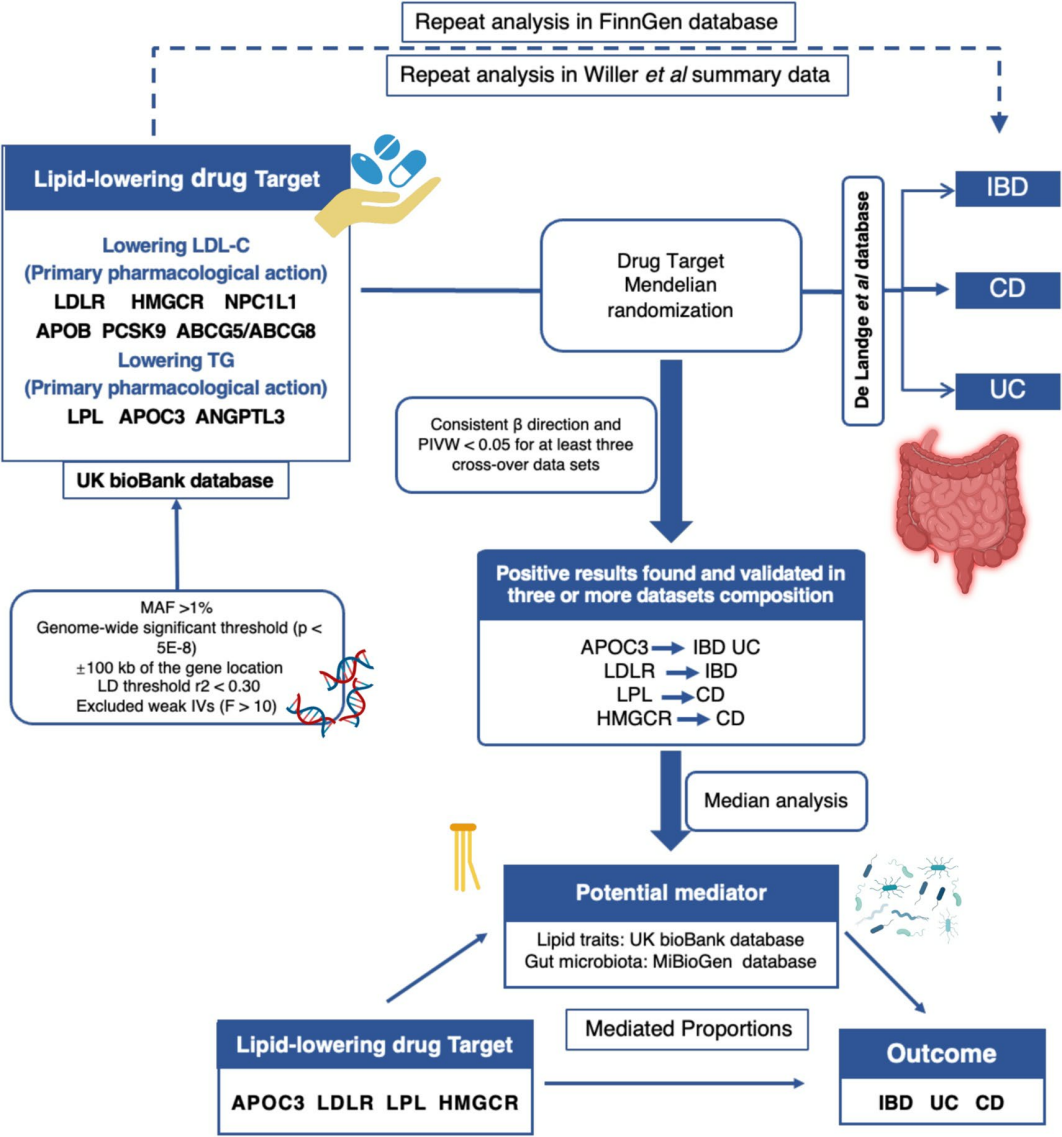
Overview of the research design. The figure was created with BioRender.com (https://biorender.com/). Abbreviations: IBD: inflammatory bowel disease; CD: Crohn’s disease; UC: ulcerative colitis; MR: Mendelian randomization; LDL-C, low-density lipoprotein cholesterol; TG, triglyceride; MAF: minor allele frequency

Figure 1 illustrates the study design. Table S1 provides comprehensive information regarding the origins of all the datasets utilized. The (STROBE MR) reporting guidelines were followed throughout the study [18]. Ethical approval was previously obtained for the utilization of data from publicly available GWAS databases.

### Instrumental variable selection

Recent protocols for managing dyslipidemia have guided the choice of lipid-lowering agents [19], with eight common lipid-lowering agents and novel therapies and two key regulatory targets selected (Table 1). The genes encoding these drug targets were recognized through the go.drugbank.com and associated publications [20–22] (Table 1).

**Table 1:** Characteristics of Lipid-lowering drug target genes

Drug class	Related Drugs	Drug targets	Primary pharmacological action	Target genes	Gene region (GRCh3.p13 by NCBI Gene)
Key regulator	RGX-501^a^	LDL Receptor	Reduced LDL-C	*LDLR*	chr19: 11200139–11244496
HMG-CoA reductase inhibitors	Lovastatin Simvastatin Atorvastatin Rosuvastatin Pravastatin Fluvastatin	HMG-CoA reductase		*HMGCR*	chr5: 74632993–74657941
Cholesterol absorption inhibitors	Ezetimibe	Niemann-Pick C1-like protein 1		*NPC1L1*	chr7: 44552134–4458092
Proprotein convertase subtilisin/kexin type 9 inhibitors	Alirocumab Evolocumab	Proprotein convertase subtilisin/kexin type 9		*PCSK9*	chr1: 55505221–55530525
Antisense oligonucleotide targeting ApoB-100 mRNA	Mipomersen	Apolipoprotein B-100		*APOB*	chr2: 21224301–21266945
Bile acid sequestrants	Colesevelam Colestipol Cholestyramine	ATP Binding Cassette Subfamily G Member 5/ATP Binding Cassette Subfamily G Member 8		*ABCG5/ ABCG8*^b^	chr2: 44039611–44065978/44066110–44110127
Key regulator		Lipoprotein Lipasea	Reduced TG	*LPL*	chr8: 19796764–19824770
peroxisome proliferator receptor alpha activators	Fenofibrate Gemfibrozil Bezafibrate Clofibrate	Peroxisome proliferator-activated receptor-a		*PPARA*	chr22:46546429–46639653
Angiopoietin-like 3 Inhibitor	Evinacumab	Angiopoietin-related protein 3		*ANGPTL3*	chr1: 63063191–63071984
Antisense oligonucleotide targeting ApoC-III mRNA	Volanesorsen	Apolipoprotein C-III		*APOC3*	chr11: 116700623–116703788

*Abbreviation*: *chr* chromosome, *mRNA* messenger ribonucleic acid, *LDL-C* low-density lipoprotein cholesterol, *TG* triglyceride

^a^RGX-501 is not yet approved for marketing and is being studied for the treatment of homozygous familial hypercholesterolemia

^b^Drug targets of bile acid sequestrants were not specified in the DrugBank. They were identified from a previous study [20]

To substitute for exposure to lipid-lowering agents, single-nucleotide polymorphisms (SNPs) positioned within the corresponding drug target (± 100 kb) and at significant levels of its downstream target substances triglycerides (TGs) or low-density lipoprotein cholesterol (LDL-C) were selected. Because apolipoprotein B (APOB) encapsulates TG as well as LDL-C to form particles [23], common SNPs possessing an effect allele frequency (EAF) exceeding 1%, associated with the APOB region, were selected to replace exposure to lipid-lowering drugs. The primary analysis was conducted using the UK Biobank database, which contains the most lipid data [24]. An external validation was conducted utilizing the data from the Global Lipid Genetics Consortium [25] (Willer et al.). The LD matrix tools (https://ldlink.nci.nih.gov/) were utilized to further ensure that no drug target gene was strongly correlated with any instrumental variable (IV) (R^2^ < 0.30).

To test this association, each variant’s potency was accessed using F-statistic. To confirm the choice of IVs for drug targeting, MR analysis of coronary artery disease (CAD) as the outcome were conducted as positive-control (Figure S1). Because the drug target gene ANGPTL3 was not detected in the positive control (CAD) cohort, this gene was excluded from further evaluation, although the results were consistent with previous findings [26]. Nine drug target genes were ultimately included in the analysis: ABCG5/ABCG8, APOC3, APOB, LDLR, LPL, HMGCR, PPARA, PCSK9, and NPC1L1. PPARA was the only gene identified as having genetic variants in the UK Biobank database.

Independent genetic variants corresponding to lipids were taken from the UK Biobank database for mediation analysis, and those corresponding to IBDs were taken from the largest sample-size summary data for IBDs provided by de Lange et al. [27] for reverse causality analysis. SNPs with significant *P* values (< 5 × 10 ^−8^) were chosen, and SNPs were removed from linkage disequilibrium with the use of a criterion (r^2^ = 0.001, clump window: 10,000 kilobases). SNPs missing EAFs were supplemented via the PhenoScanner website. Gut microbiota-associated SNPs were obtained from the MiBioGen consortium [28], a database for which 72.3% of the included participants are individuals of European ancestry. To select the appropriate SNPs, the significance level was compromised (*P* < 5 × 10 ^−5^; unidentified bacterial taxa were excluded). All SNPs were removed from linkage disequilibrium with the use of a criterion (r^2^ = 0.001, clump window: 10,000 kilobases).

### Outcomes IBD database

The main analysis was performed with the De Lange et al. summary database, while data from FinnGen (inngen.fi/fi/hyodynna_tuloksia) were utilized for external validation. To prevent duplication of sample data sources, the data from UK Biobank were not extracted separately.

### Statistical analysis

To guarantee that each IV was aligned with the identical effect allele, exposure and outcome data were reconciled before analysis [29]. The exposures were associated with the outcomes (*P* > 5 × 10^–8^) and the presence of palindromic SNPs. The principal analysis method was Inverse-variance weighting (IVW) [30]. MR-Egger regression [31], median [32], and pattern-based [33] were used for assessing reliability. Consistency in the obtained directions using these methods was deemed a strong indication of a robust effect.

An IVW model with multiplicative random effects was used for SNPs with heterogeneity, which were detected using Cochran's Q. In addition, a reverse causality test was performed using the MR Steiger test [34]. This approach posits that for valid IVs, the difference in exposure should be greater than the difference in outcome. In order to assess relative pleiotropy, The MR‒Egger intercept tests and MR pleiotropy residual and outlier (MR-PRESSO) were employed [35]. A horizontal *P* -index of 0.05 was used to detect outlier SNPs using the MR-PRESSO outlier test. MR results were assessed using the leave-one-out method to check their robustness.

A network MR analysis was executed to check whether the obtained associations were direct. For lipids, multivariate MR was also performed, and multivariate IVW was used as the primary method. TG, LDL-C, and APOB were evaluated within Model 1 to identify the main factor responsible for the causal associations between lipid-lowering drug targets and IBDs because APOB encapsulates TG and LDL-C to create particles. HDL-related phenotypes were assessed for an association with IBDs by analyzing HDL-C and APOA1 in Model 2. A reverse causality analysis was also performed to explore whether IBDs cause dyslipidemia. For significant associations, there may be potential mediating effects (exposure-mediation-outcome pathway). To this end, the coefficient product method was used to evaluate the indirect effect, and indirect effects' standard errors were determined using the delta method [36].

To account for multiple testing of 9 drug targets, Bonferroni-corrected significance levels, utilizing a P-value cutoff below 0.0056 (0.05/9), were employed. Statistical significance was considered to be indicated by an observed *P* < 0.05 for additional analyses. Statistical analysis was conducted using R packages, including TwoSampleMR and MendelianRandomization.

## Results

### Drug proxy IVs and IBD risk

Table S2 displays the SNPs that were considered in the drug-target analyses for every region. All the SNPs had F statistics greater than 29, minimizing potential weak instrumental bias. Table S3 presents the genetic instrument strength and statistical power of the MR analysis for each drug target (> 80% power at α = 0.05).

Table 2 shows the correlation between the genetic proxies of the drug targets and IBD risk. According to the IVW MR data, there were strong inverse correlations of the genetically predicted TG level modified by APOC3 with IBD risk (Odds Ratio [OR], 95% Confidence Interval [CI]): 0.87 (0.80–0.95); *P* = 0.0023) and UC risk (0.83 (0.73–0.94); *P* = 0.0028), as well as a suggestive linkage to CD (OR: 0.90 (0.81–0.99); *P* = 0.035). These results indicate that APOC3 inhibitors might increase susceptibility to IBDs (comprising CD and UC). There was some evidence that there was a connection between genetic mimicry resulting from HMGCR inhibition and a greater risk of IBD and CD (OR_IBD_ = 0.72, *P* = 0.0087; OR_CD_ = 0.68, *P* = 0.018). An analogous pattern was noted for a protective effect of the genetically predicted LDL level modified by LDLR on IBD risk (OR: 1.18 (1.03–1.36); *P* = 0.018). Convincing data was obtained indicating an increase in the genetically predicted LDLR resulting in a decreased risk of CD (OR: 1.40 (1.17–1.67); *P* = 2.60E-04). The protective effects against IBD risk (OR: 1.20 (1.09–1.33); *P* = 1.70E-04) and CD risk (OR: 1.26 (1.11–1.43); *P* = 2.60E-04) were similar when the genetic variation in TG levels was modified by LPL. There are indications that NPC1L1 inhibition is associated with a lower risk of IBDs (OR: 1.94 (1.04–3.61);* P* = 0.037), whereas PCSK9 inhibition might increase CD risk (OR: 0.73 (0.56–0.96); *P* = 0.022).

**Table 2:** Mendelian randomization results of lipid-lowering drug genetic variants in target genes from the UK Biobank database with risk of IBD from the database of de Lange et al.

Drug Target	Methods	IBD		UC		CD	
		**OR (95% CI)**	***P* value**	**OR (95% CI)**	***P* value**	**OR (95% CI)**	***P* value**
ABCG5 (APOB)	WM	0.78 (0.54- 1.14)	0.2	0.82 (0.53- 1.26)	0.37	0.61 (0.36- 1.03)	0.07
	IVW	0.84 (0.64- 1.12)	0.23	0.92 (0.66- 1.31)	0.66	0.83 (0.52- 1.30)	0.41
ABCG5 (LDL)	WM	0.77 (0.54- 1.09)	0.14	0.85 (0.56- 1.30)	0.46	0.62 (0.38- 1.02)	0.06
	IVW	0.83 (0.64- 1.09)	0.19	0.93 (0.68- 1.27)	0.64	0.79 (0.52- 1.22)	0.29
APOB (LDL)	WM	1.07 (0.89- 1.28)	0.47	0.98 (0.75- 1.27)	0.86	1.09 (0.87- 1.36)	0.46
	IVW	0.98 (0.86- 1.12)	0.75	0.88 (0.71- 1.08)	0.22	1.07 (0.91- 1.25)	0.44
APOB (APOB)	WM	0.99 (0.85- 1.15)	0.9	0.93 (0.77- 1.13)	0.47	0.98 (0.82- 1.17)	0.8
	IVW	0.97 (0.88- 1.08)	0.62	0.87 (0.75- 1.00)	0.05	1.07 (0.94- 1.22)	0.32
APOC3 (APOB)	WM	0.59 (0.39- 0.90)	1.50E-02	0.46 (0.26- 0.82)	8.40E-03	0.78 (0.48- 1.27)	0.32
	IVW	0.52 (0.38- 0.69)	9.90E-06	0.41 (0.27- 0.63)	5.10E-05	0.67 (0.46- 0.97)	3.50E-02
APOC3 (TG)	WM	0.89 (0.79- 1.01)	0.06	0.90 (0.78- 1.04)	0.16	0.94 (0.82- 1.07)	0.35
	IVW	0.87 (0.80- 0.95)	2.30E-03	0.83 (0.73- 0.94)	2.80E-03	0.90 (0.81- 0.99)	3.70E-02
HMGCR (APOB)	WM	0.69 (0.47- 1.00)	5.00E-02	0.74 (0.44- 1.25)	0.26	0.57 (0.36- 0.90)	1.50E-02
	IVW	0.68 (0.52- 0.91)	8.70E-03	0.76 (0.45- 1.29)	0.31	0.64 (0.44- 0.92)	1.50E-02
HMGCR (LDL)	WM	0.70 (0.50- 0.97)	3.10E-02	0.73 (0.47- 1.12)	0.15	0.64 (0.42- 0.98)	3.80E-02
	IVW	0.72 (0.56- 0.92)	8.70E-03	0.76 (0.52- 1.11)	0.15	0.68 (0.50- 0.94)	1.80E-02
LDLR (APOB)	WM	1.16 (0.95- 1.43)	0.15	0.90 (0.69- 1.17)	0.43	1.53 (1.16- 2.01)	0.0026
	IVW	1.20 (1.04- 1.38)	1.10E-02	0.89 (0.74- 1.08)	0.24	1.43 (1.19- 1.71)	1.30E-04
LDLR (LDL)	WM	1.17 (0.95- 1.43)	0.14	0.90 (0.70- 1.16)	0.43	1.48 (1.14- 1.94)	0.0036
	IVW	1.18 (1.03- 1.36)	1.80E-02	0.90 (0.75- 1.07)	0.24	1.40 (1.17- 1.67)	2.60E-04
LPL (APOB)	WM	1.95 (1.11- 3.43)	2.00E-02	1.50 (0.72- 3.12)	0.27	2.42 (1.18- 4.95)	1.60E-02
	IVW	2.10 (1.35- 3.28)	1.10E-03	1.64 (0.93- 2.90)	0.09	2.55 (1.44- 4.53)	1.30E-03
LPL (TG)	WM	1.17 (1.02- 1.33)	2.00E-02	1.08 (0.92- 1.28)	0.33	1.19 (1.01- 1.41)	4.00E-02
	IVW	1.20 (1.09- 1.33)	1.70E-04	1.12 (0.99- 1.27)	0.06	1.26 (1.11- 1.43)	2.60E-04
NPC1L1 (APOB)	WM	1.85 (0.78- 4.39)	0.17	1.64 (0.56- 4.76)	0.37	2.50 (0.80- 7.86)	0.12
	IVW	2.15 (0.93- 4.99)	0.07	2.04 (0.84- 4.94)	0.12	2.07 (0.65- 6.61)	0.22
NPC1L1 (LDL)	WM	1.68 (0.84- 3.34)	0.14	1.65 (0.67- 4.06)	0.28	2.84 (1.07- 7.59)	3.70E-02
	IVW	1.94 (1.04- 3.61)	3.70E-02	1.91 (0.92- 3.96)	0.08	1.63 (0.65- 4.11)	0.3
PCSK9 (APOB)	WM	1.00 (0.76- 1.31)	0.98	1.13 (0.79- 1.61)	0.5	0.75 (0.52- 1.09)	0.13
	IVW	0.87 (0.70- 1.07)	0.18	1.01 (0.78- 1.29)	0.96	0.72 (0.54- 0.95)	2.00E-02
PCSK9 (LDL)	WM	1.00 (0.76- 1.31)	0.98	1.13 (0.80- 1.60)	0.49	0.76 (0.53- 1.08)	0.12
	IVW	0.88 (0.72- 1.07)	0.2	1.02 (0.80- 1.30)	0.87	0.73 (0.56- 0.96)	2.20E-02
PPARA (TG)	Wald ratio	2.20 (0.41–11.85)	0.36	3.25 (0.38–27.68)	0.28	2.40 (0.28- 20.91)	0.43

*WM* Weighted median method, *IVW* Inverse variance weighted method

Additional IVs were selected based on the APOB GWAS summary data for these drug targets. These results were consistent, except that the NPC1L1 inhibition in IBD patients was not significantly different (Table 2). The correlation trends were similar among the other MR methods (Table 2).

Neither MR-PRESSO nor MR‒Egger intercept tests revealed evidence of pleiotropy (Table S4). The outcomes from the leave-one-out assessments additionally substantiated the robustness of these results (Figure S2).

### External validation of drug proxy IVs and IBD risk

**Fig. 2:**
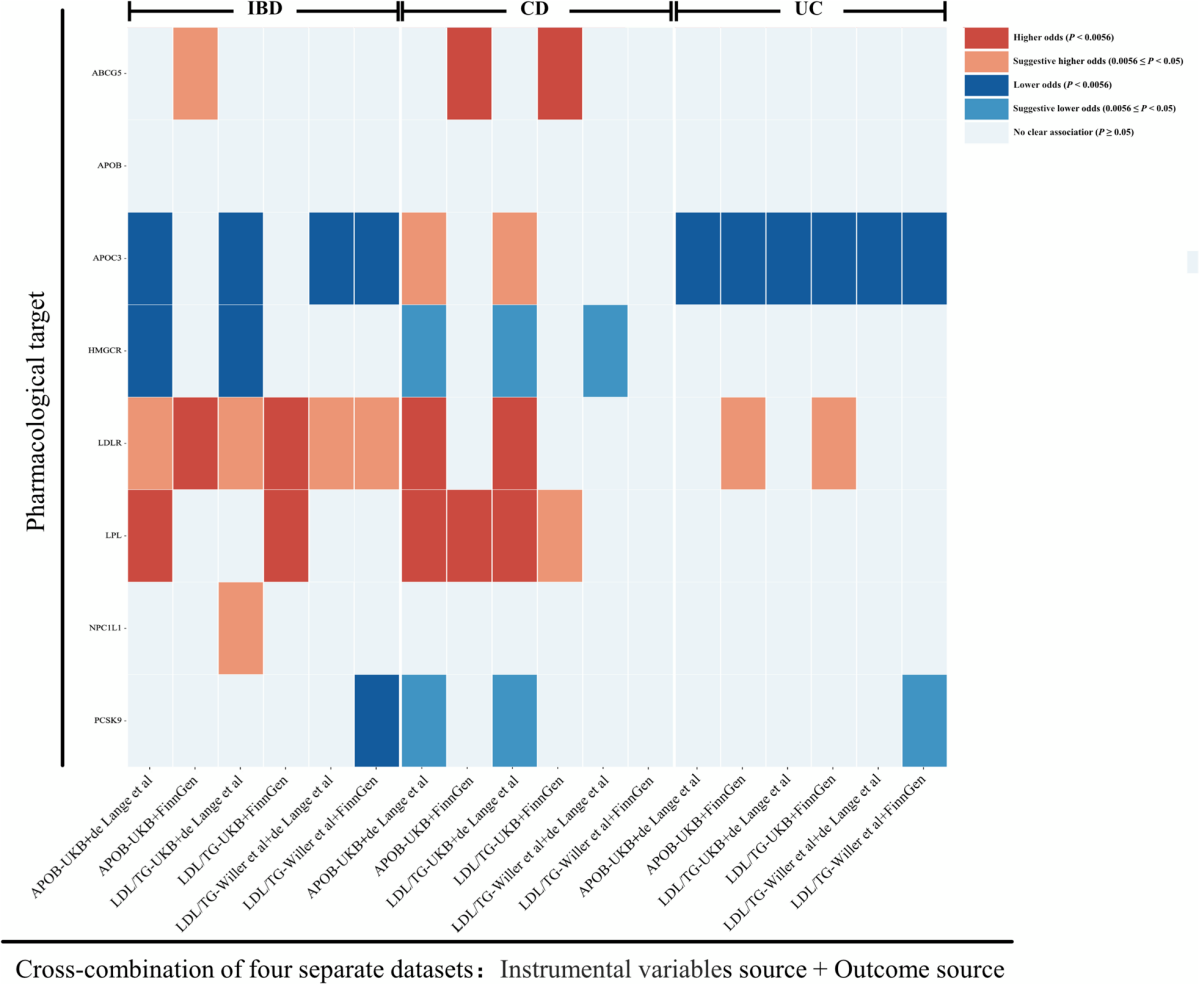
Combined MR findings for genetic mimicry of lipid-lowering medication targets and IBD risk in patients in different dataset combinations. The abscissa represents the IV source database combined with the IBD source database.T

The MR analyses were further repeated on variant data from different datasets (from the UK Biobank [24] and Willer et al. [25]) and on outcome data from different datasets (from de Lange et al. [27] and FinnGen). The associations of the genetically predicted TG level modified by APOC3 and the genetically predicted LDL level modified by LDLR with IBDs were replicated across all the investigated dataset combinations. The genetically predicted LDL level modified by HMGCR from different sources was associated with only CD according to the pooled data from de Lange et al. However, the genetically predicted TG/APOB level modified by LPL from only the UK Biobank dataset was associated with CD. The relationship of the genetically inferred TG/APOB level modified by APOC3 with UC was replicated in all database combinations. The association of genetically predicted TG/APOB levels modified by APOC3 with UC was consistent across all investigated dataset combinations (Fig. 2; Table S5). No pleiotropic effects were detected (Table S6).

### Mediation analysis

#### Lipid traits and IBD risk

Given that IBDs are commonly associated with dyslipidemia according to observational studies, dyslipidemia may mediate the effect of lipid-lowering agents on IBD risk. To avoid any potential pleiotropic effects, outlier SNPs were removed, and ultimately, 104 to 202 SNPs were retained for IBDs (including CD and UC) (Figure S3). Because all lipids (TG, LDL-C, HDL, APOB or APOA1) exhibited significant heterogeneity for IBDs (comprising CD and UC), the random-effects inverse-variance weighted (IVW) approach was employed. Among the five lipids, solely TG exhibited a negative association with IBDs (OR: 0.87 (0.87–0.96); *P* = 0.004). Multivariate MR analysis revealed no association between TG levels and IBDs (Figure S4). No causal effects were found between TG concentrations and CD (OR: 0.96 (0.86–1.08); *P* = 0.476) or UC (OR: 0.92 (0.82–1.03); *P* = 0.133). No associations were found between LDL-C, HDL, APOB or APOA1 and IBDs (comprising CD and UC) (Figure S3). The levels of five lipids were not causally affected by IBDs (including CD and UC) according to bidirectional MR analysis (Table S7).

#### The gut microbiota and IBD risk

Considering the significant role of the microbiota in the onset of IBDs, further exploration was conducted to determine whether these gut microbiota are mediators of the influence of lipid-modifying medications on IBD susceptibility. Gut microbiota causally associated with IBDs were initially detected (Table S8), after which the impact of lipid-reducing agents on these associated strains were tested (Table S9). Finally, indirect effects and fractions of mediation were calculated using the technique of product-of-coefficients (Associations with direct and indirect effects in opposite directions were excluded) (Table 3). *Clostridium *sensu stricto 1, *Clostridiaceae* 1, and *Lachnospiraceae* FCS020 group partially mediated the total effect of APOC3 inhibition on IBDs (proportion mediated: 17.39% (95% CI: 14.34–20.44%), *P* = 7.30E-03; 20.06% (95% CI: 16.91–23.22%), *P* = 2.70E-03; 12.05% (95% CI: 10.08–14.01%), *P* = 0.033; respectively) (Table 3). *Lactobacillaceae* partially mediated the total effect of LDLR enhancement on IBDs (proportion mediated: 17.83% (95% CI: 12.16–23.5%), *P* = 0.046) (Table 3).

**Table 3:** Mediation analysis of the effect of lipid-lowering agents on IBD risk via potential mediators

Exposure	Adjustment	Outcome	Indirect effects estimate (95% CI)	*P* Value	Median proposition (%) (95%CI)
APOC3	*Family_* *Clostridiumsensustricto*1	IBD	-0.03(-0.06, 0)	7.30E-03	17.39(14.34–20.44)
APOC3	*Family_* *Clostridiaceae*1	IBD	-0.03(-0.06, -0.01)	2.70E-03	20.06(16.91–23.22)
APOC3	*Group_* *LachnospiraceaeFCS020group*	IBD	0.017(0, 0.04)	3.34E-02	12.05(10.08–14.01)
LDLR	*Family_* *Lactobacillaceae*	IBD	0.04(0.01, 0.09)	4.55E-02	17.83(12.16–23.5)
APOC3	*Family_* *Clostridiumsensustricto*1	UC	-0.028(-0.06, 0.04)	0.061	14.77(10.46–19.08)
APOC3	*Order_Bacillales*	UC	-0.031(-0.07, 0.04)	0.081	15.01(9.67–20.35)
LPL	*Group_* *RuminococcaceaeUCG009*	CD	0.03(0, 0.07)	0.064	13.67(9.93–17.41)

*IBD* Inflammatory bowel disease, *CD* Crohn’s disease, *UC* Ulcerative colitis

## Discussion

APOC3 inhibition increases susceptibility to IBD and UC, while HMGCR inhibition is a risk factor for CD. LDLR and LPL are promising targets for treating IBD and CD, respectively. These findings were validated in two independent IBD datasets generated by constructing different genetic instruments using two independent lipid datasets. Mediation analysis suggested that the negative effect of APOC3-mediated TG levels on IBDs may occur partly through a decrease in TG, decrease in *Clostridiaceae* 1, increase in *Clostridium *sensu stricto 1, or increase in the *Lachnospiraceae* FCS020 group. The protective effect of LDLR-mediated LDL-C levels on IBDs may be partly mediated through increasing *Lactobacillaceae* abundance. Nonetheless, it is important to mention that the effects of drugs on the overall gut microbiota [37] were not considered.

One study reported that APOC3 expression was decreased in IBD patients [13]. However, investigations on the association of APOC3 with IBD risk, particularly UC, have been limited. Furthermore, mediation analysis suggested that the harmful effects of APOC3 on IBDs might be mediated in part by lowering TG levels, decreasing *Clostridiaceae* 1 levels, increasing *Clostridium *sensu stricto 1 levels, or increasing the *Lachnospiraceae* FCS020 group. The incidence of UC is greater in patients with lower serum TG levels, but lower serum TG levels do not affect the incidence of CD [38]. These findings indicated an inverse correlation with the risk of IBD when examining TG levels, yet no such link was observed with either CD or UC risk, and the correlation became null after multivariable MR adjustment for LDL and APOB. However, the correlation between TG levels and IBD risk needs to be further explored. These findings indicate that genetically predicted TG levels modified by APOC3 are linked to a higher susceptibility to IBD and UC. Therefore, monitoring for potential adverse effects on IBDs (UC) is necessary in individuals treated with volanesorsen (an antisense oligonucleotide that targets ApoC3 mRNA).

These insights could have substantial implications for the development of therapeutic strategies. Specifically, a nuanced understanding of the role of APOC3 in modulating TG levels and IBD risk can inform the design of additional targeted therapies. For instance, this could lead to the development of more refined APOC3-targeting therapies that minimize the risk of IBDs, providing a safer treatment profile for patients. Future animal models could be instrumental in elucidating the precise mechanisms by which APOC3 impacts IBD risk, allowing for the preclinical assessment of these targeted therapies. Subsequently, clinical trials could be tailored to the monitoring of IBD outcomes, particularly UC, in patients treated with APOC3 modulators, such as volanesorsen. This approach would enable the careful balancing of therapeutic benefits in lipid management against potential gastrointestinal side effects.

No effect of lipid characteristics on CD risk reduction was found in this study, indicating that the contribution of LPL augmentation to CD susceptibility does not depend on its lipid-lowering capacity. The absence of causal connections of TG, PPARA, and APOC3 with IBDs implies that regulating LPL might have other physiological functions in addition to TG metabolism. Many clinical drugs, such as statins, metformin, thiazolidinediones and ω-3 fatty acids, have pleiotropic effects on LPL [39]. Moreover, metformin is thought to reduce the risk of IBDs or improve IBD risk [40, 41], although no study has focused on the effect of these drugs on IBDs through the action of LPL. An observational study of 197 patients with IBD reported increased LPL expression in those patients [10], but that study included patients taking statins and steroids. It has been reported that statins can increase the level of LPL expression [42–44]. This study, which involved drug target MR analysis, revealed the inverse association between LPL and CD risk, and clinical trials or basic research may be useful for evaluating the role of LPL activators in CD.

Most studies of lipid-lowering agents in IBD patients have focused on statins. However, the impact of statins on the susceptibility to IBDs (including CD and UC) is still inconclusive. The current meta-analysis did not provide evidence to substantiate a significant correlation between the use of statins and incident CD or UC [8, 14]. In fact, besides possessing anti-inflammatory properties, statins also exhibit proinflammatory actions [45]. Soh et al. demonstrated that subjects with a record of statin consumption experienced a higher occurrence of CD [38], which is consistent with this conclusion. However, certain research points to a negative association between statin usage and the development of new-onset CD [9, 46]. Lochhead et al. [9] found that lipophilic statins, but not hydrophilic statins, had a correlation with CD risk. Furthermore, past statin use did not show a link to CD risk, and the strongest inverse association was observed among current statin users [9]. This also suggested that the duration of statin exposure may have an impact on the association with IBD risk. The few available observational studies may be subject to confounding factors; for example, many studies have shown a notable disparity in the prevalence of CAD and dyslipidemia among individuals with IBDs compared to that in the control group. However, the findings reflected lifetime exposure (clinical drug effects are usually short-term), the confidence intervals were wide (indicating that the possibility of feasibility was not high; 0.05 < *P* < 0.0056), and the association between HMGCR (the targets of statins) and CD was only suggestive, which was not overlooked. Future rigorous epidemiological research is required to explore the relationship between statin use and CD.

### Study strengths and limitations

This study's merits are highlighted by its design and potential impact on future therapeutic strategies. This study employed MR to reduce the impact of confounding factors commonly observed in observational research. Furthermore, genetic variations, which occur at the early stages of life, can be used to determine whether an exposure precedes disease onset. This temporal information is essential for inferring causal direction, offering clarity in the understanding of disease etiology. Additionally, this study used MR to elucidate how biomarker alterations affect long-term health outcomes through targeted therapeutic interventions. This approach is pivotal in forecasting the potential effectiveness of drugs and pinpointing any adverse reactions associated with specific drug targets. The current study revealed that inhibition of APOC3 correlates with a heightened risk for IBD and UC, while blocking HMGCR emerges as a risk determinant for CD. The study also highlights LDLR and LPL as prospective targets for IBD and CD and could inform the development of targeted treatment options.

Several limitations must be considered when analyzing the research findings. First, this research relies on genetic variants that representing the lifelong effects of lipid level fluctuations throughout life on the susceptibility to IBDs, which may differ significantly from the influences of short-duration lipid-modifying treatments. The main advantage of MR analysis is the identification of causal associations rather than the estimation of the actual strength of associations [47]. Second, this study focused on the presence or absence of IBDs but not on the specific courses of IBDs [48]. For patients with IBDs, phases of remission and relapse often alternate, and the specific timing of disease onset is often difficult to predict. Despite the importance of finding genomic markers related to IBD activity, identifying such markers under the current research conditions is still a challenging task. Exploration via MR analysis was not possible due to the absence of GWAS data on the course of IBDs. Third, although this study successfully predicted the potential effects of several drug targets, possible off-target effects were not assessed. Finally, the results might not be fully applicable to diverse populations due to the use of a mainly European sample. However, this limitation also has the advantage of minimizing the bias induced by population stratification.

## Conclusion

In conclusion, this study underscores potential links between lipid-lowering drug targets and IBDs. APOC3 inhibition increases susceptibility to IBD and UC, while HMGCR inhibition is a risk factor for CD. LDLR and LPL have emerged as promising targets for treating IBD and CD, respectively.

This study provides evidence with clinical implications for IBD management, highlighting the need for caution when using APOC3 inhibitors such as volanesorsen in individuals with IBD, considering the possible increased adverse event risk. The correlation between HMGCR inhibition and a heightened risk of CD suggested re-evaluating statin prescriptions for these individuals. Additionally, the research points to LDLR and LPL as promising drug targets for IBD and CD and could lead to alternative treatments, especially for those unresponsive to current options. These effects seem to be partially independent of lipid-lowering effects, indicating new paths for drug development.

Thus, while providing new insights, this research also highlights the need for further investigations into the intricate relationships among lipid metabolism, genetics, and IBDs. Clinical trials should assess the therapeutic viability of modulating these targets in IBD patients, while animal research is required to dissect the mechanisms of these genetic associations. These findings offer a potential direction for further research, emphasizing the need to consider the broader impact of lipid metabolism on IBD pathophysiology.

Overall, the findings suggest that lipid metabolism plays a potential role in IBDs, suggesting a need for personalized treatment strategies based on genetic and metabolic profiles.

### Supplementary Information

**Additional file 1: Table S1.** Phenotype descriptions and distributions. **Table S2A.** Characteristics of lipid-lowering genetics variants in target genes from UK Biobank database. **Table S2B.** Characteristics of lipid-lowering genetics variants in target genes from Willer et al. **Table S3.** Statistical power estimates for drug-target MR analyses. **Table S4.** Heterogeneity and pleiotropy tests of instrument effects (drug targets from UK Biobank database). **Table S5.** External validation of the causal relationship between lipid-lowering drug genetic variants and IBD IVW-MR analysis in different datasets combinations. **Table S6.** Heterogeneity and pleiotropy tests of instrument effects for external validation. **Table S7.** Association of genetically proxied inflammmatory bowel diseases with risk of lipids traits. **Table S8.** Causal effects and heterogeneity and pleiotropy tests of genetically predicted gut microbiota on inflammatory bowel diseases. **Table S9.** The relationship between genetic mimicry of lipid-lowering drugs that can affect Inflammatory bowel disease and the microbiota that can affect Inflammatory bowel disease.

**Additional file 2: Figure S1.** IVW-MR association between nine lipid-lowering drug targets and coronary heart disease. **Figure S2.** Leave-one -out plot of MR analyses from lipid-lowering drugs targets to inflammatory bowel disease in databases with a *P* value of less than 0.05 for IVW-MR results. **Figure S3.** Association between genetically predicted lipid traits and the risk of Inflammatory bowel disease. **Figure S4.** Multivariable Mendelian randomization using the inverse- variance weighted method. 

## Data Availability

Data is provided within the manuscript or supplementary information files.
