# Supplementary material for: Correlations between genetically predicted lipid-lowering drug targets and inflammatory bowel disease
Source: Lipids Health Dis. 2024 Jan 29;23:31. doi: 10.1186/s12944-024-02026-y (PMC10823737; doi:10.1186/s12944-024-02026-y)

Supplementary figure legends

Figure S1 IVW-MR association between nine lipid-lowering drug targets and coronary heart disease.

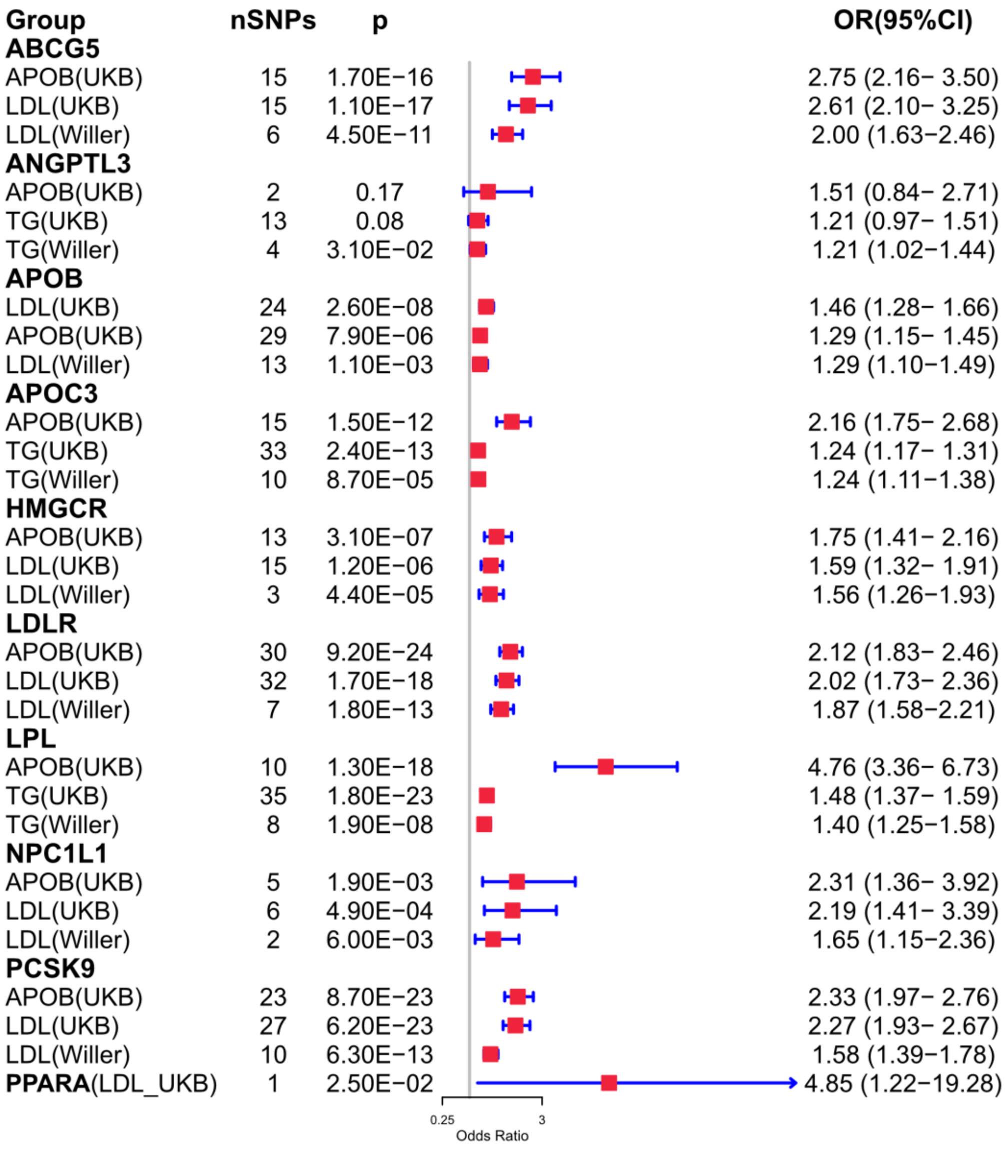

Figure S2 Leave-one-out plot of MR analyses from lipid-lowering drugs targets to inflammatory bowel disease in databases with a P value of less than 0.05 for IVW-MR results.

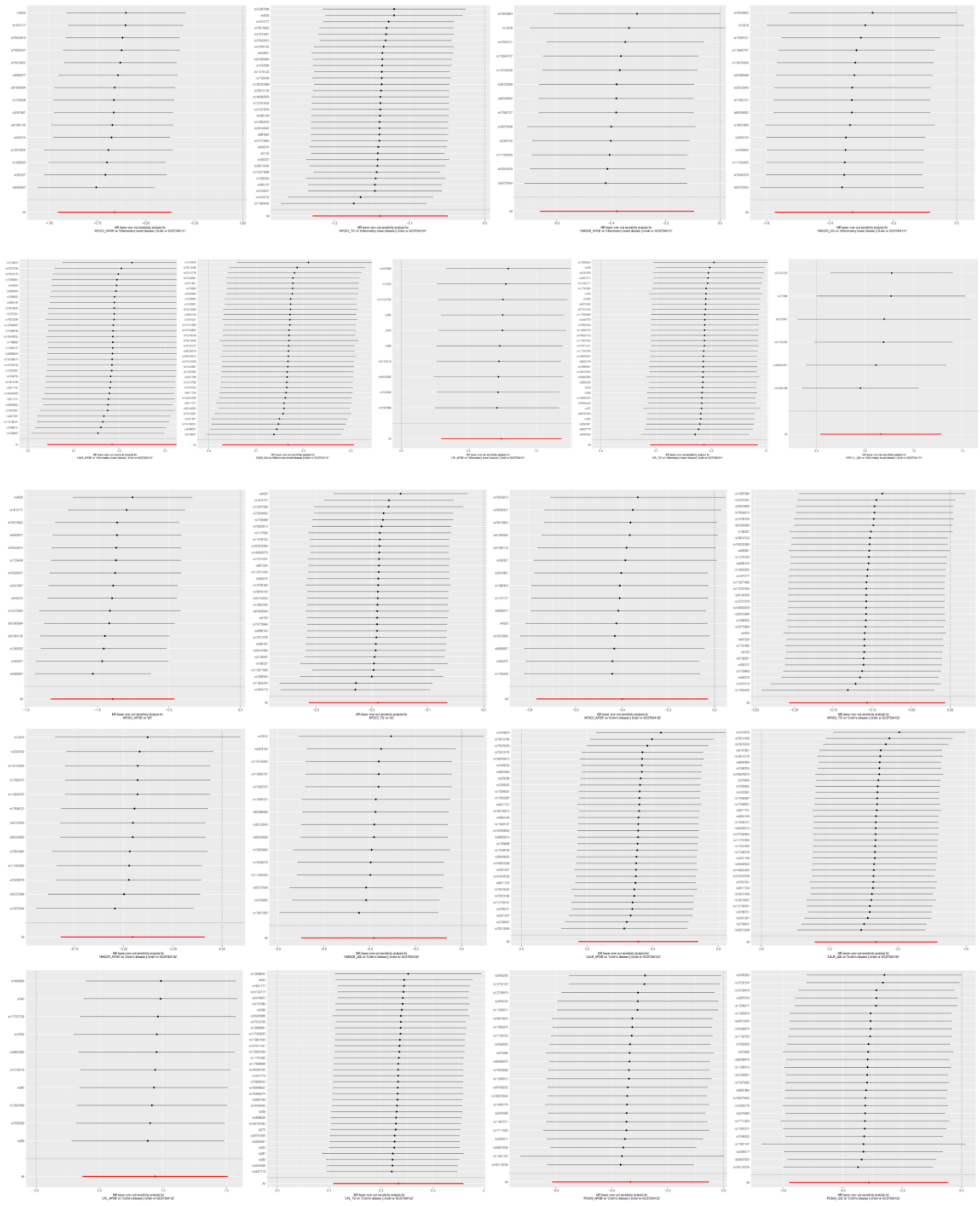

Figure S3 Association between genetically predicted lipid traits and the risk of Inflammatory bowel disease.

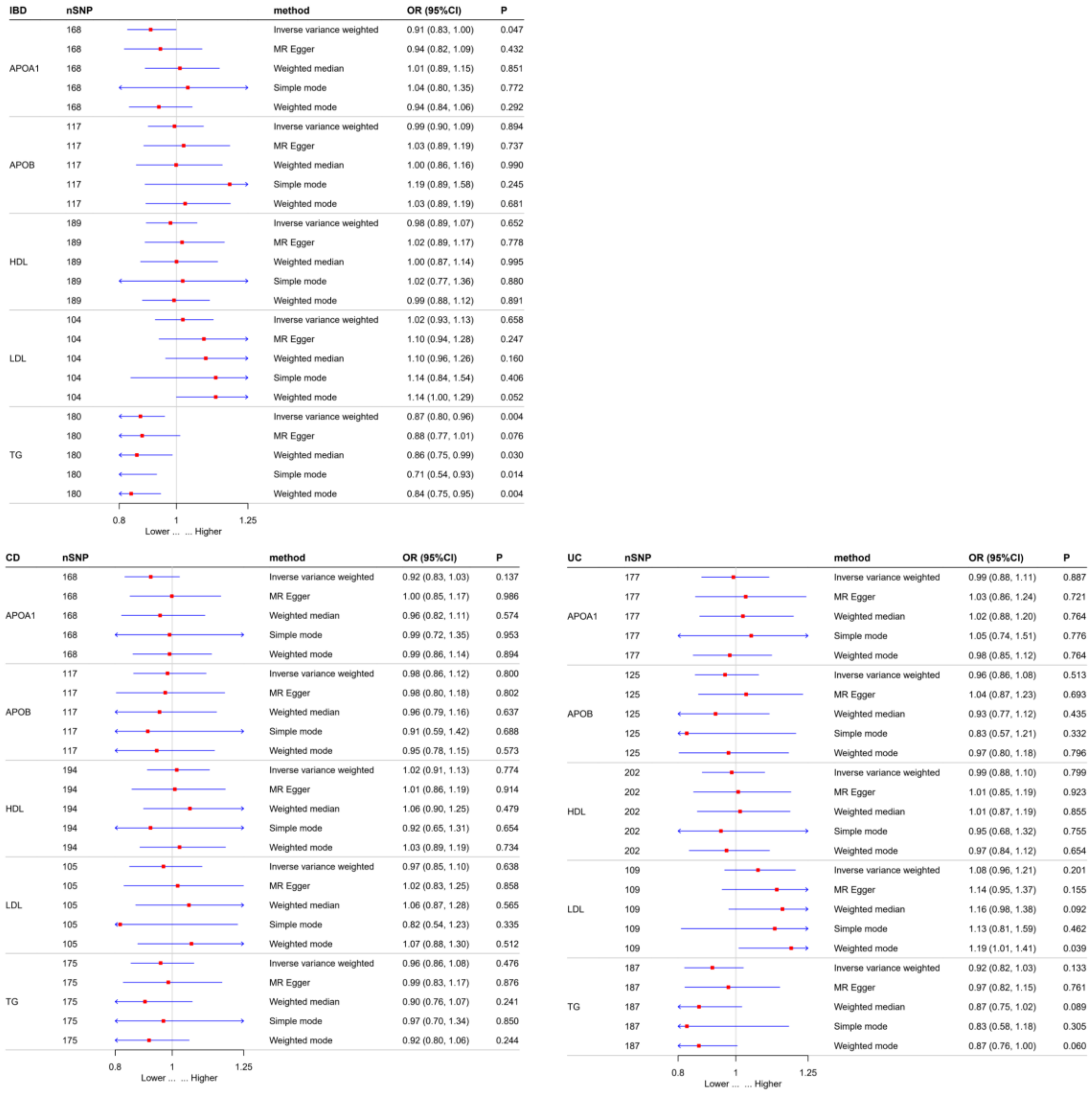

Figure S4 Multivariable Mendelian randomization using the inverse-variance weighted method. Model 1 included apolipoprotein B, low-density lipoprotein cholesterol and triglycerides. Model 2 included apolipoprotein A1 and high-density lipoprotein cholesterol.

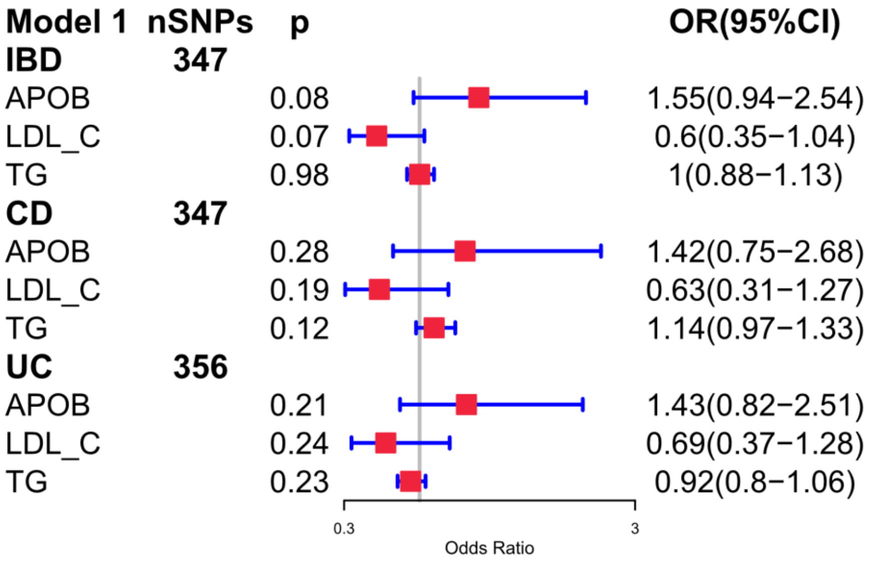

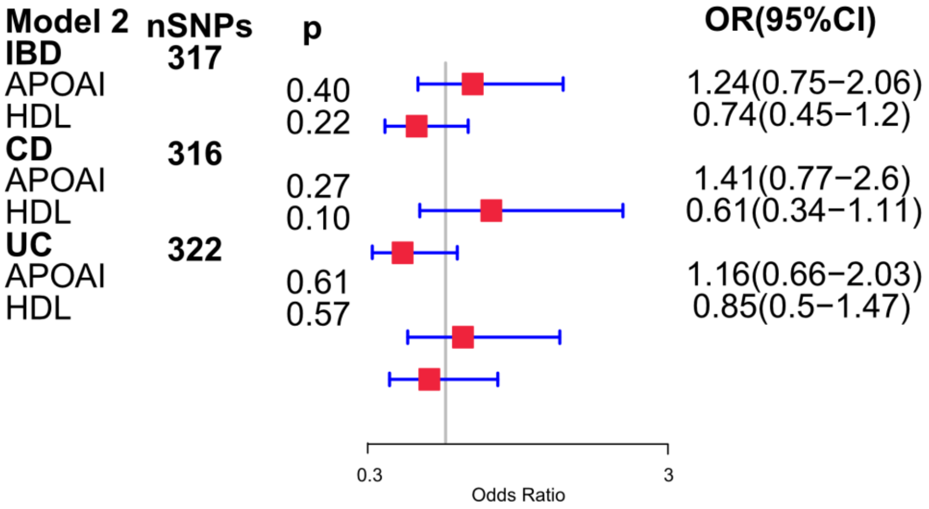

Supplement: Supplementary file 2 — Additional file 2: Figure S1. IVW-MR association between nine lipid-lowering drug targets and coronary heart disease. Figure S2. Leave-one -out plot of MR analyses from lipid-lowering drugs targets to inflammatory bowel disease in databases with a P value of less than 0.05 for IVW-MR results. Figure S3. Association between genetically predicted lipid traits and the risk of Inflammatory bowel disease. Figure S4. Multivariable Mendelian randomization using the inverse- variance weighted method. [file 12944_2024_2026_MOESM2_ESM.pdf]
